# Gravitropic response in radiata pine seedlings. Searching molecular keys

**DOI:** 10.1186/1753-6561-5-S7-P161

**Published:** 2011-09-13

**Authors:** Patricio Ramos, Nicolas Cruz, Alvaro González, Raúl Herrera

**Affiliations:** 1 Laboratorio de Fisiología Vegetal y Genética Molecular, Instituto de Biología Vegetal y Biotecnología, Universidad de Talca, Chile

## 

**Figure 1:**
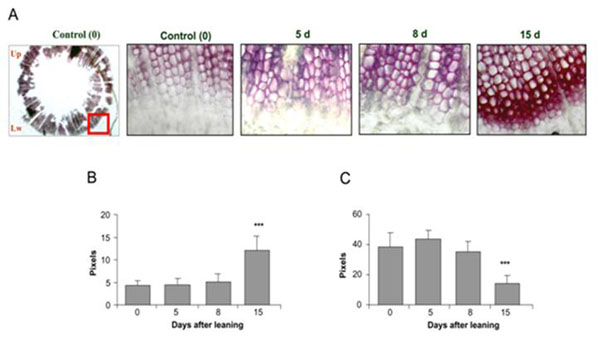
Anatomical modification on lignin deposition and xylem cell shape on inclined seedlings.

Coniferous trees develop compression wood in response to gravitropic stimuli. In nature this response can be generated by growth in slope, exposure to snow or high winds [1]. However, genes and molecular mechanism involved in this phenomenon are still unknown. We studied gene expression in response to gravitropic stimulation induced by 45° inclination in *Pinus radiata* D. Don one year old seedlings. To characterize the gravitropic response, whole seedlings were inclined and transversal cuts were performed in order to identify morphological wood characteristics. Xylem cells were visualized by optical microscopy in a time course experiment (fig. 1). On the other hand, a transcriptomic approach was assayed generating libraries based on the Suppressive Subtractive Hybridization (SSH) strategy [2]. This technique allows the isolation of genes differentially expressed between two samples. The libraries were generated from total RNA extracted at 2.5, 10, 24 hours and 30 days from the inferior and superior stem half. The sequences obtained were assembled, analyzed and ontology classified by biological process, molecular function and cell components. The information give clue about the molecular mechanism related to this phenomenon. To validate the differential gene expression by qPCR analyses, housekeeping genes were evaluated in order to have normalization genes for the gravitropic stress response. We could identify a large number of genes activation involved in different initial process, previous to the anatomical hallmarks of compression wood formation.

One-year old *Pinus radiata* seedlings were inclined during 15 days (d) and transversally cut at 5d, 8d and 15d. Stem slices were stained using a solution of phloroglucinol (2A). From these preparations, wall thickness (2B) and cell diameter (2C) of xylem cells were measured.
